# The effect of language on performance: do gendered languages fail women in maths?

**DOI:** 10.1038/s41539-021-00087-7

**Published:** 2021-04-06

**Authors:** Tamar Kricheli-Katz, Tali Regev

**Affiliations:** 1 Buchman Faculty of Law, Tel Aviv University, Tel Aviv-Yafo, Israel; 2 Tiomkin School of Economics, Interdisciplinary Center Herzliya, Herzliya, Israel

**Keywords:** Social sciences, Interdisciplinary studies

## Abstract

Research suggests that gendered languages are associated with gender inequality. However, as languages are embedded in cultures, evidence for causal effects are harder to provide. We contribute to this ongoing debate by exploring the relationship between gendered languages and the gender gap in mathematics achievements. We provide evidence for causality by exploiting the prominent (but not exclusive) practice in gendered languages of using masculine generics to address women. In an experiment on a large representative sample of the Hebrew-speaking adult population in Israel, we show that addressing women in the feminine, compared to addressing them in the masculine, reduces the gender gap in mathematics achievements by a third. These effects are stronger among participants who acquired the Hebrew language early in childhood rather than later in life, suggesting that it is the extent of language proficiency that generates one’s sensitivity to being addressed in the masculine or in the feminine. Moreover, when women are addressed in the masculine, their efforts (in terms of time spent on the maths test) decrease and they report feeling that “science is for men” more than when addressed in the feminine. We supplement the analysis with two experiments that explore the roles of general and task-specific stereotypes in generating these effects.

## Introduction

Languages vary by whether they require speakers to grammatically mark gender. In gendered languages such as French, Spanish, German, and Hebrew, parts of speech—pronouns, nouns, adjectives, and/or verbs—have feminine and masculine forms. In addition, in such languages, forms of speech that refer to one gender only are used more frequently than they are in gender-neutral languages. Generic use of the masculine form for both females and males is more prominent in gendered languages than in gender-neutral ones^1,2^.

Research suggests that gendered languages are associated with gender inequality. Studies have shown that the countries in which gendered languages are spoken tend to be associated with greater gender inequality in labor, credit, board membership, division of household labor, and education than are countries whose languages feature gender-neutral grammatical systems^3–10^. Other studies demonstrate experimentally that addressing people in languages with grammatical gender affects their attitudes, perceptions, and motivations. In one study, answering a survey about sexist attitudes in a language with grammatical gender (French or Spanish) was found to increase the reported sexist attitudes, compared to answering the same survey in English^11^. In a related experimental study, addressing women in the masculine in an academic-motivation questionnaire generated lower reports of task value and intrinsic goal orientation compared with addressing women in the gender-neutral form of the language^12^. Although the literature has established cross-language correlations between gendered languages and gender inequality, and has shown the effects of gendered language on attitudes, it has not yet provided evidence for the causal effects of using a gendered language on women’s and men’s performances.

**Fig. 1:**
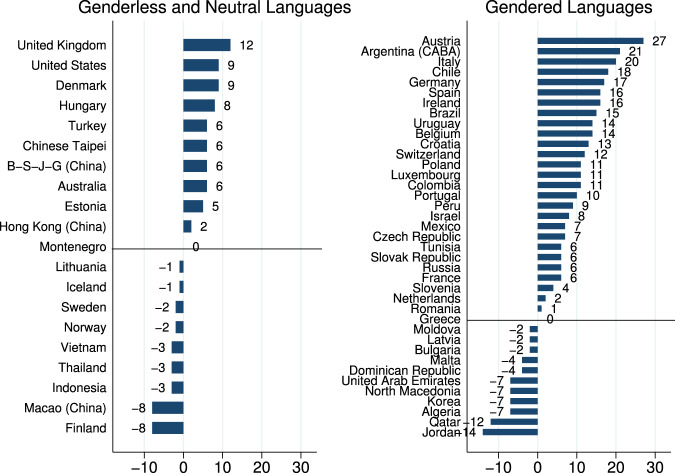
The gender gap in mathematics by country and type of language, boys–girls (PISA, 2015). The 2015 data were collected by the Programme for International Student Assessment (PISA). PISA assesses the average gender gap in mathematics achievements for 15-year-olds in OECD and OECD partner countries. The data was merged with the dataset generated by Prewitt-Freilino et al.^3^ that denotes whether the language spoken in a country is gendered genderless or gender neutral. The merged dataset includes 59 countries with an average gender gap in mathematics achievements of 4.71 (SD = 8.78). The PISA scores are scaled to fit an approximately normal distribution with a mean around 500 score points and a standard deviation around 100 score points. Thus a 1 point difference on the PISA scale corresponds to an effect size (Cohen’s *d*) of 0.01.

This study focuses on the effects of gendered languages on mathematics achievements of women and men, and explores the mechanisms generating them. The cross-country variations in the gender gap in mathematics achievement motivate the project. In Fig. 1, we present the gender gap in mathematics achievements in 2015 by whether the language spoken is gendered or not (a positive gap reflects higher scores for boys compared to girls).

The average gender gap in mathematics achievements in countries whose languages are gendered is 6.15, whereas the average gender gap in mathematics achievements in countries whose languages are genderless or gender neutral is only 1.90 (*t*-test, *p* = 0.039). In Supplementary Fig. 1, we present similar patterns (*t*-test, *p* < 0.01) for the 2018 PISA data.

Although these data provide an opportunity to observe global patterns, there might be additional factors, correlated with whether a language is gendered or not, which generate the trends in gender inequality we observe. In other words, although survey data are very useful in documenting correlations, it is difficult to use them to show a causal effect between the linguistic features and grammatical structures of languages, and between the attitudes and behaviors of the people who speak them. As languages are embedded in cultures^13–15^, it is hard to rule out the possibility that the cultural differences correlated with linguistic features and grammatical structures of languages generate the observed differences in the attitudes and behaviors of people. Nonetheless, previous studies on the effects of language on behavior tended to use survey data and explore cross-country variations^16,17^.

We take a different methodological approach. We provide evidence for causality by exploiting the prominent (but not exclusive) practice in gendered languages of using masculine generics to address women. In an experiment on a large representative sample of the Hebrew-speaking adult population in Israel, we show that addressing women in the masculine, compared to the feminine, negatively affects their performance in maths. In fact, when women are addressed in the feminine and men in the masculine, the gender gap in mathematics achievements is reduced by a third, compared to when both women and men are addressed in the masculine. These effects are stronger among participants who acquired the Hebrew language early in childhood rather than later in life. This finding suggests that it is the extent of language proficiency that generates one’s sensitivity to being addressed in the masculine or in the feminine. When women are addressed in the masculine, their efforts (in terms of time spent on the maths test) decrease and they report feeling that “science is for men” more than when addressed in the feminine. Finally, we supplement the analysis with two experiments that explore the roles of task-specific and general sex stereotypes in generating these effects.

Addressing women in the masculine in a mathematics achievement test may affect their performance, because their sense of alienation is activated. Women who are addressed in the masculine may find it harder to view themselves as the prototypical test takers than they would when addressed in the feminine. These perceptions may lead women to believe less in their ability to succeed and therefore to decrease the levels of effort, concentration, and performance they invest in the task.

Indeed, experimental studies conducted in various languages have shown that the use of linguistic masculine forms within a language (e.g., he vs. the lawyer) evokes a male bias in mental representations and makes readers or listeners think more of men than of women as exemplars of a person category^18–32^. In one recent study, it was shown that the use of gender-neutral pronouns reduces the biases in favor of traditional gender roles and categories, and generates more positive attitudes toward women and LGBT (lesbian, gay, bisexual, and transgender) individuals in public affairs^33^.

Addressing women in the masculine in a mathematics achievements test may also make gender and sex stereotypes more salient. Indeed, stereotypes and cultural beliefs about gender are easily activated; once activated, they may affect the quality of women’s performance^34,35^. In general, people hold highly defined and consensual stereotypes about who women and men are. People tend to associate communion (expressive traits) with women and agency and competence (instrumental traits) with men^36–39^. In addition to the general stereotypes and cultural beliefs about gender, people tend to hold task-specific stereotypes and cultural beliefs about the ways in which women and men perform in specific tasks^40–42^. Thus, e.g., although there is limited evidence for intrinsic biological differences in mathematical abilities between women and men^43^, men are perceived to be better than women in maths and science^44–46^.

Stereotypes and cultural beliefs about women’s lower ability in maths and science generate a “stereotype threat” and negatively affect girls’ and women’s actual performance, as well as their willingness to attribute their success to their abilities rather than to their efforts^34,35,38^. The tendency to perform worse when negative stereotypes are salient has been shown to be related to the anxiety, distraction, and decreased efforts^47–50^ caused by the perceived lower expectations by others^34,51^ and lower expectations of oneself^52^. Indeed, stereotypes about women and maths are so easily activated that merely asking women to indicate their gender before taking a maths test negatively affected their performance^53^.

To provide evidence for a causal relationship between the gendering of the language and the gender gap in mathematics achievements, we asked a large random representative sample of the adult Hebrew-speaking population in Israel to complete an SAT-type maths exam online when addressed in the feminine or in the masculine. Such exams are designed to measure a high school student’s readiness for college. Hebrew is a gendered language in which the grammatical rule is to use the masculine form of the language as generic for both females and males^54^. In fact, when the gender of the recipient is unknown or when addressing a group that consists of more than one man (regardless of the number of women in the group), the rule is to use the masculine form. In the experiment, we used the masculine singular and the feminine singular forms of the Hebrew language (by varying the form of the verb “answer” in the instructions).

When women take online surveys in Hebrew and their gender is known, they sometimes encounter the use of feminine generics and sometimes of masculine ones. Thus, this setting enabled us to use both forms (the masculine and the feminine) without worrying that one form would not feel natural for female test takers. Men, however, are very rarely addressed in the feminine in Hebrew. The online setting also eliminates peer group effects that may interact with the effects of being addressed in the masculine or in the feminine. In particular, we did not want women participants to be bothered by whether the men in the lab are being addressed in the feminine or in the masculine.

In the experiment, participants were asked to take an SAT-type maths test comprising six questions. The experiment consisted of two experimental conditions (addressing participants in the feminine or in the masculine). Questions were taken from the qualitative reasoning sections of previous university entrance exams, published on the official website of the Israeli National Center for Testing and Evaluation. These questions are designed to assess the ability of participants to use numbers and mathematical concepts to solve quantitative problems. Throughout the tests, both female and male participants were addressed either in the feminine or in the masculine forms. Unlike in the actual psychometric exam, we gave no time limitations for the maths tests. Upon completion, participants were asked to fill out an implicit association test^55^, followed by an explicit bias questionnaire addressing their attitudes and beliefs about the associations of women and men with the sciences and the liberal arts. Finally, participants were asked to report their own attitudes toward science. The demographic characteristics were originally obtained by the survey company when participants were recruited for the panel.

The initial sample for this experiment included 963 participants. Eighteen percent were born outside of Israel. We included them in the sample, because many of them spoke Hebrew from early childhood. In our analysis, we utilized their age at immigration to Israel as a proxy for their language proficiency^56^. We hypothesized that the effects of being addressed in the masculine compared to the feminine weakens as participants’ proficiency in the language decreases (because they are less sensitive to the gendering of the language). Thus, we predicted that the effects of being addressed in the masculine compared to the feminine, will be smaller when the age at immigration to Israel is greater.

## Results

### The gender gap in mathematics achievements

Table 1 presents the descriptive statistics for the variables we use in the analysis. We calculated participants’ maths grades by summarizing the number of their correct answers (see Supplementary Fig. 2 for the distribution of scores). On average, participants scored 63% on the maths test. Only 79.85% of the participants completed the full questionnaire, which included a gender-science Implicit Association Test (https://implicit.harvard.edu/) and the attitudes and explicit biases survey.

**Table 1:** Descriptive statistics.

Variable	Native Hebrew speakers		Immigrants		All		Min	Max
	Mean	SD	Mean	SD	Mean	D.		
Demographics								
Women	0.50		0.53		0.51			
Age	41.91	15.53	49.16	16.36	43.45	15.97	18.00	74.00
Higher education	0.72		0.73		0.72			
Above average income	0.20		0.24		0.21			
Immigration age	0.00	0.00	13.38	12.21	2.41	7.30	0.00	62.00
Immigrant from the Former USSR	0.00		0.25		0.05			
Political affiliation—left	0.30		0.31		0.30			
Political affiliation—center	0.20		0.25		0.21			
Political affiliation—right, other	0.50		0.44		0.49			
Exam outcomes								
Maths grade	0.63	0.30	0.63	0.31	0.63	0.30	0.00	1.00
Maths time (in minutes)	6.29	4.68	7.05	5.65	6.45	4.91	0.45	49.86
*N*	759		204		963			

**Fig. 2:**
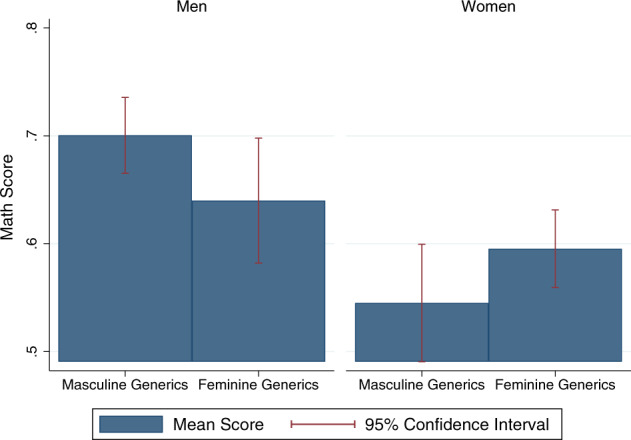
Mean scores in maths, by gendered address. Female participants received higher scores when addressed in the feminine, compared to the masculine. Male participants received higher scores when addressed in the masculine, compared to the feminine.

Figure 2 graphically presents native Hebrew speakers’ mean scores in maths by gender and experimental condition.

On average, women received lower scores than men received. Whereas the average score of women was 57.8, the average score of men was 68.0 (*t*-test, *p* < 0.001, *N* = 759). However, addressing participants in the feminine form resulted in higher scores for women and with lower scores for men. Whereas the average score of women who were addressed in the masculine was 54.5, the average score of women who were addressed in the feminine was 59.5 (*t*-test, *p* = 0.059, *N* = 383). Although the average score of men who were addressed in the masculine was 70.1, the average score of men who were addressed in the feminine was 64.0 (*t*-test, *p* = 0.032, *N* = 376).

To assess the statistical significance of our results, we ran ordinary least squares (OLS) regression models predicting the effect of addressing participants in the feminine form on their test performance. Results are presented in Table 2. In all the models, the sample includes only native Hebrew speakers who were born in Israel, unless otherwise specified (*N* = 759). When addressed in the masculine, women received scores that were lower by 15 percentage points than the scores men received (Model 1; F(1,755) = 19.08, *p* < 0.001, *N* = 759). When men were addressed in the feminine, their scores were lower by 6.9 percentage points than when addressed in the masculine (Model 1; F(1,755) = 3.20, *p* = 0.074, *N* = 759). When addressed in the masculine, women’s maths scores were lower by 5.1 percentage points (−6.9 + 12.0), relative to when addressed in the feminine (Model 1; F(1,755) = 2.31, *p* = 0.129). However, when men were addressed in the feminine, their scores were lower by 6.9 percentage points than when addressed in the masculine (Model 1; F(1,755) = 3.20, *p* = 0.074, *N* = 759). In fact, when both women and men are addressed in the feminine, the gender gap in mathematics achievements becomes statistically nonsignificant. When women were addressed in the feminine and men in the masculine, women received scores that are lower only by 9.9 percentage points on average than men’s (Model 1; F(1,755) = 13.90, *p* < 0.001, *N* = 759).

**Table 2:** OLS regression models predicting grades in maths.

	(1)	(2)	(3)
Female	−0.150***	−0.154***	−0.153***
	(0.034)	(0.033)	(0.033)
Feminine generics	−0.069*	−0.077**	−0.061*
	(0.038)	(0.038)	(0.036)
Female × Feminine generics	0.120**	0.139***	0.107**
	(0.051)	(0.050)	(0.048)
Age		−0.001	
		(0.001)	
Higher education		0.073***	
		(0.025)	
Above average income		0.065**	
		(0.030)	
Political party fixed effects		Y	
Immigrant			0.068**
			(0.033)
Immigration age			−0.005
			(0.004)
Female × Feminine generics × Immigration age			−0.011*
			(0.007)
Female × Immigration age			0.007
			(0.005)
Feminine generics × Immigration age			0.007
			(0.005)
Constant	0.698***	0.655***	0.700***
	(0.019)	(0.038)	(0.019)
N	759	759	926
Adjusted *R*^2^	0.029	0.066	0.032

SEs in parentheses; Regression (3) includes immigrants; *p < 0.1, **p < 0.05, ***p < 0.01.

When the demographic characteristics of participants are accounted for, the aforementioned effects all become statistically significant and remain relatively similar in magnitude. Thus, e.g., when addressed in the masculine, women’s maths scores were lower by 6.14 (−7.75 + 13.89) percentage points relative to when addressed in the feminine (Model 2; F(1,737) = 3.4, *p* = 0.066, *N* = 759).

Model 3 includes immigrants and controls for their immigration age. The effects are stronger among participants who acquired the Hebrew language early in childhood rather than later in life. Being a year older when immigrating decreases the effect of feminine generics on women by 1.1 percentage point (F(1, 917) = 3.00, *p* = 0.083, *N* = 926). This finding suggests that it is the extent of proficiency in Hebrew that generates one’s sensitivity to being addressed in the masculine or in the feminine in the experiment. Moreover, as sensitivity to the gendering of a language might be affected by whether one’s native language is gendered or not, we expected the effects of being addressed in the feminine to be greater for immigrants from countries whose languages are gendered compared to immigrants from countries whose languages are not gendered. Indeed, the differences in participants’ scores do suggest that being addressed in the feminine in Hebrew has a greater negative effect on female participants who immigrated from countries whose languages are gendered. Yet, the very small sample size^17^ of female participants who have immigrated from countries whose languages are not gendered does not enable us to make statistical inferences.

It is important to note that some of the participants did not answer all the questions in their tests. The grades we use as dependent variables in the main analysis (Table 2) were calculated by treating missing answers as wrong answers. For robustness, we test our predictions on: (1) the subsample of participants who answered all the maths questions and (2) the full sample but with a dependent variable (grade) that is calculated as the number of correct answers out of the number of questions answered. The results of the two robustness tests are similar in magnitude and statistical significance to the results we report in the main analysis (see Supplementary Table 1).

### Efforts

**Fig. 3:**
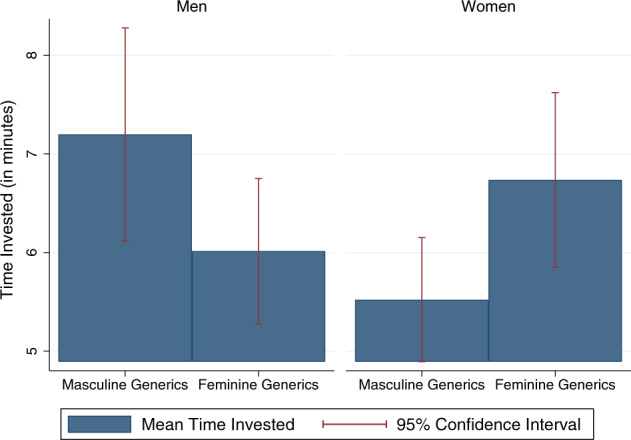
Mean time invested in the maths test, by gendered address. Female participants spent more time on their maths exam when addressed in the feminine, compared to the masculine. Male participants spent less time on their maths exam when addressed in the feminine, compared to the masculine.

We use the time participants spent on the maths test as a proxy for their efforts and motivation to succeed. The time was reordered for each question that the participants answered. The sample therefore includes only the participants who provided answers to all the questions in their tests. Indeed, the time participants spent on the maths test correlated with the score they attained (a correlation of 0.24). We therefore tested whether the time participants spent on their maths test was affected by whether they were addressed in the feminine or in the masculine. In Fig. 3, we graphically present participants’ mean time invested in the maths test, by gender and experimental condition (for native Hebrew speakers only).

Addressing participants in the feminine form increased the time women spent on their maths test and decreased the time men did. In fact, when comparing women and men who were both addressed in the masculine, we find that women spent significantly less time on their maths test relative to men (*t*-test, *p* = 0.019). However, there was no statistical difference in the time women and men spent on their maths test when women and men were both addressed in the feminine (*t*-test, *p* = 0.296).

Similar results are found in OLS regression models predicting the time participants spent on their maths test (Supplementary Table 2). When addressed in the masculine, women spent on average 1.87 min less on the test than men did (model 1; F(1,684) = 6.15, *p* = 0.013, *N* = 688). When addressed in the feminine, women spent 1.18 min more on the maths test than when addressed in the masculine (an increase of 0.3 SDs, model 1, F(1,684) = 4.59, *p* = 0.033, *N* = 688). When men were addressed in the feminine, they spent 1.44 min less on the maths test compared to when they were addressed in the masculine (model 1; F(1,684) = 3.48, *p* = 0.063, *N* = 688). Accounting for the demographic characteristics of participants left the effects practically unchanged (Model 2).

### Prototypical test takers and sense of alienation

After completing the experiment, participants were asked (on a scale of 1 to 7) to what extent they agreed with the statement that “science is for men.” As predicted, when addressed in the feminine, participants reported agreeing less with the statement that “science is for men.” Whereas when addressed in the masculine, the average response of participants was 5.03; when addressed in the feminine, the mean was 4.84 (*t*-test, *p* = 0.007). No statistically significant differences were found between the effects on female and male participants. Participants were also asked to what extent they agreed with the statement that “arts and humanities are for women.” No statistically significant differences were found between the responses of participants who were addressed in the masculine and the responses of those who were addressed in the feminine. Similar effects were found in regression models (Supplementary Table 3). These findings suggest that gendered languages affect perceptions of who the prototypical test taker is and the sense of alienation: when women and men are addressed in the feminine in an SAT-type maths test, they view women as more representative of the prototypical scientist, compared to when addressed in the masculine.

Lastly, no statistically significant differences were found between the results of the Implicit Association Tests of participants who were addressed in the masculine and the responses of those who were addressed in the feminine. This finding is compatible with studies that have shown that implicit associations are immediate and unconscious, and are therefore very difficult to change^30^.

### Supplementary experiments

#### Positive gender stereotypes

Whereas gender stereotypes and cultural beliefs about women are negative in some contexts (those associated with competence and performance in maths and sciences), they tend to be positive in others (those associated with warmth and care-giving)^25^. Here, unlike in the main experiment, we use an assignment that is associated with positive stereotypes about women and with negative stereotypes about men (an assignment that is stereotypically associated with women strengths and men’s weaknesses). If indeed addressing women in the masculine makes gender stereotypes and cultural beliefs more salient, then we would expect the positive stereotypes to lead women to perform better in an assignment that is associated with positive stereotypes about women and negative stereotypes about men.

The assignment we use here is a reading comprehension test about empathy.

Altogether, 690 people participated in the experiment (335 women and 345 men). They were all randomly assigned to being addressed in the masculine or in the feminine (altogether, 333 participants were addressed in the masculine and 347 in the feminine). We find that in this reading comprehension test about empathy, women performed better than men. Whereas the average score of women was 53.7, the average score of men was 50.2 (*t*-test, *p* = 0.058; *N* = 680).

As predicted, women performed better when addressed in the masculine compared to when addressed in the feminine. When addressed in the feminine, women’s average score was only 51, whereas when addressed in the masculine the average score of women was 56.6 (*t*-test, *p* = 0.042). The differences for men were statistically nonsignificant. These findings suggest that being addressed in the masculine evokes gender stereotypes and cultural beliefs, and thus affects performance.

#### Gender-neutral assignments

Finally, we seek to investigate the effects of being addressed in the masculine on women and men, when the assignment is relatively gender-neutral, so that gender-specific stereotypes are irrelevant. Based on the literature on general sex stereotypes^25^, we predict that women will perform worse when addressed in the masculine, compared to the feminine—even when tasks are relatively gender neutral.

The assignment we use here is a word association test. Participants were asked to write as many words as they could in the span of 60 s. The words had to begin with consecutive letters of the alphabet (a word that begins with the letter “a,” followed by a word that begins with the letter “b”, etc.). Participants were told that they would be scored based on accuracy and on the total length of all the words they provide. Female and male participants were addressed either in the feminine or in the masculine.

Altogether, 674 people participated in this experiment (334 women and 340 men). They were randomly assigned to being addressed in the masculine (*N* = 343) or the feminine (*N* = 331). On average, women performed better than men in this assignment. Whereas the average total number of letters women provided was 42.8, the average number of letters that men provided was only 39.6 (*t*-test, *p* = 0.035). As predicted, being addressed in the masculine negatively affected women’s performance in this relatively gender-neutral task. When addressed in the masculine, the average number of letters women provided was 40.8, whereas when addressed in the feminine it was 45 (*t*-test, *p* = 0.058). The average number of letters men provided was 38.2 when addressed in the masculine and 41.2 when addressed in the feminine. This gap was statistically nonsignificant (*t*-test, *p* = 0.202).

## Discussion

In an experiment on a large representative sample of the Hebrew-speaking adult population in Israel, we find that addressing women in the masculine negatively affect their performance in maths. These effects are stronger among participants who acquired the Hebrew language early in childhood rather than later in life. When women are addressed in the masculine, their efforts (in terms of time spent on the maths test) decrease and they report feeling that “science is for men” more than when addressed in the feminine. Not surprisingly, the findings about the effects of addressing men in the feminine are weaker and less robust, probably because of the salience of sex stereotypes of men’s greater competence in maths. We supplement the analysis with two experiments. The first supplementary experiment provides evidence for the role of task-specific stereotypes in affecting women’s performance: we show that when tasks are stereotypically associated with women’s strengths, women perform better when addressed in the masculine compared to when addressed in the feminine. The second experiment provides evidence for the possible roles of general stereotypes in affecting women’s performance: we show that when tasks are relatively gender neutral, women perform better when addressed in the feminine compared to when addressed in the masculine.

We make two main contributions in this study: first, we provide experimental evidence for the powerful role of language in affecting how people behave. Whereas most previous studies have shown correlations between the usage of certain languages and between economic behavior and market outcomes, here we provide evidence for a causal relation between the use of a language and people’s performance. Second, building on the literature on “stereotype threat^34,35^,” our findings suggest that stereotypes and cultural beliefs about sex are so deeply embedded in languages that they unconsciously impact people’s beliefs, efforts, and performance in ways that reinforce gender inequality and thus further legitimize and sustain gender inequality.

Our findings have some limitations. Most notably, the participants in the studies took the tests at home—individually, not in a classroom. This setup was chosen so as to study the effects of being addressed in the feminine on performance net of other environmental effects. Yet, the gender composition of the test takers in a classroom, e.g., is one factor that may interact with the effects of being addressed in the feminine on performance: in a mixed-gender classroom, women may feel discomfort with being addressed in the feminine, if they know that men in the classroom are also addressed in the feminine. Even when women do not know whether men are addressed in the feminine or in the masculine, their performance may still be affected, because they may spend time and effort thinking about how men in the classroom are being addressed.

It is also noteworthy that the effects on men of being addressed in the feminine are negative in some contexts, although smaller than the effects on women and marginally significant. It follows, therefore, that to improve women’s performance, women and men should be granted the right to choose whether they wish to be addressed in the feminine or in the masculine in exams. It is also worth noting that because the stereotypes and cultural beliefs embodied in the language may affect women’s learning experiences and not only their performance in exams, the language spoken in classrooms should also be modified to include feminine generics and neutral forms.

Naturally, modifying the languages of exams, and even the language spoken in classrooms, would not eliminate altogether the gender gaps in maths and reading comprehension performance. Gender inequality is persistent and over-determined: it is consistently and simultaneously generated and maintained in multiple spheres of life and spanning different levels of analysis^40^. Yet, tackling such inequality within each realm or level of analysis is important in generating the possibility for change.

## Methods

The participants for the study were recruited by Dialogue, a survey company that specializes in Internet-based surveys and uses a representative panel of the Israeli population. The initial sample for this experiment included 963 participants (491 women and 472 men) who were randomly assigned to one of the two experimental conditions. Out of the 963 participants, 18% were born outside of Israel. 490 participants took a maths test while being addressed in the masculine and 473 took a maths test while being addressed in the feminine.

All materials used in the experiments, as well as their English translation, are provided in the Supplementary Materials section in the Supplementary Information file.

### Ethical compliance declaration

We have complied with all relevant ethical regulations with regard to human research participants. Approval for the study protocols was obtained from the institutional review boards of the Interdisciplinary Center Herzliya and Tel Aviv University. Written informed consent was obtained from all human participants.

### Reporting summary

Further information on research design is available in the Nature Research Reporting Summary linked to this article.

## Supplementary information

Supplementary Information

Reporting Summary

## Data Availability

Data are available on Open-ICPSR: Inter-university Consortium for Political and Social Research [distributor], 2020-11-10, 10.3886/E126081V2.

## Code availability

Code is available on Open-ICPSR: Inter-university Consortium for Political and Social Research [distributor], 2020-11-10, 10.3886/E126081V2.
