# Supplementary material for: The effect of language on performance: do gendered languages fail women in maths?
Source: NPJ Sci Learn. 2021 Apr 6;6:9. doi: 10.1038/s41539-021-00087-7 (PMC8024272; doi:10.1038/s41539-021-00087-7)
Supplement: Supplementary file 1 — Supplementary Information [file 41539_2021_87_MOESM1_ESM.pdf]

Supplementary Information

Supplementary Tables:

Supplementary Table 1: OLS Regression Models Predicting Grades in Math, Robustness

	Only participants who answered all questions	Grades based on answered questions only
	(1)	(2)
Female	-0.128***	-0.127***
	(0.028)	(0.029)
Feminine Generics	-0.051*	-0.054*
	(0.029)	(0.032)
Female X Feminine Generics	0.080*	0.082*
	(0.041)	(0.044)
Age	0.074***	0.080***
	(0.022)	(0.022)
Higher Education	0.077***	0.083***
	(0.023)	(0.025)
Above Average Income	0.686***	0.670***
	(0.021)	(0.022)
Political Party Fixed Effects	Y	Y
Constant	0.686***	0.670***
	(0.021)	(0.022)
N	675	705
Adjusted R^2	0.099	0.085

Standard errors in parentheses; * p<0.1 ** p<0.05 *** p<0.01

Supplementary Table 2: OLS Regression Models Predicting Time Invested in Math

	(1)	(2)
Female	-1.871**	-1.906**
	(0.755)	(0.775)
Feminine Generics	-1.440*	-1.512*
	(0.772)	(0.811)
Female X Feminine Generics	2.624***	2.645***
	(0.949)	(0.966)
Age		0.038***
		(0.015)
Higher Education		1.345**
		(0.545)
Above Average Income		0.249
		(0.610)
Political Party Fixed Effects		Y
Constant	7.405***	4.888***
	(0.684)	(0.804)
N	688	688
Adjusted R^2	0.006	0.004

Standard errors in parentheses; * p<0.1 ** p<0.05 *** p<0.01

Supplementary Table 3: OLS Regression Models Predicting "Science Is for Men"

	(1)	(2)
Female	-0.314**	-0.300**
	(0.148)	(0.150)
Feminine Generics	-0.283*	-0.232
	(0.157)	(0.168)
Female X Feminine Generics	0.286	0.212
	(0.220)	(0.228)
Age		-0.006
		(0.004)
Higher Education		0.241*
		(0.133)
Above Average Income		0.090
		(0.139)
Political Party Fixed Effects		Y
Constant	5.087***	5.142***
	(0.090)	(0.196)
N	617	617
Adjusted R^2	0.007	0.012

Standard errors in parentheses; * p<0.1 ** p<0.05 *** p<0.01

Supplementary Figures:

Supplementary Figure 1: The Gender Gap in Mathematics by Country and Type of Language, Boys-Girls (PISA, 2018)

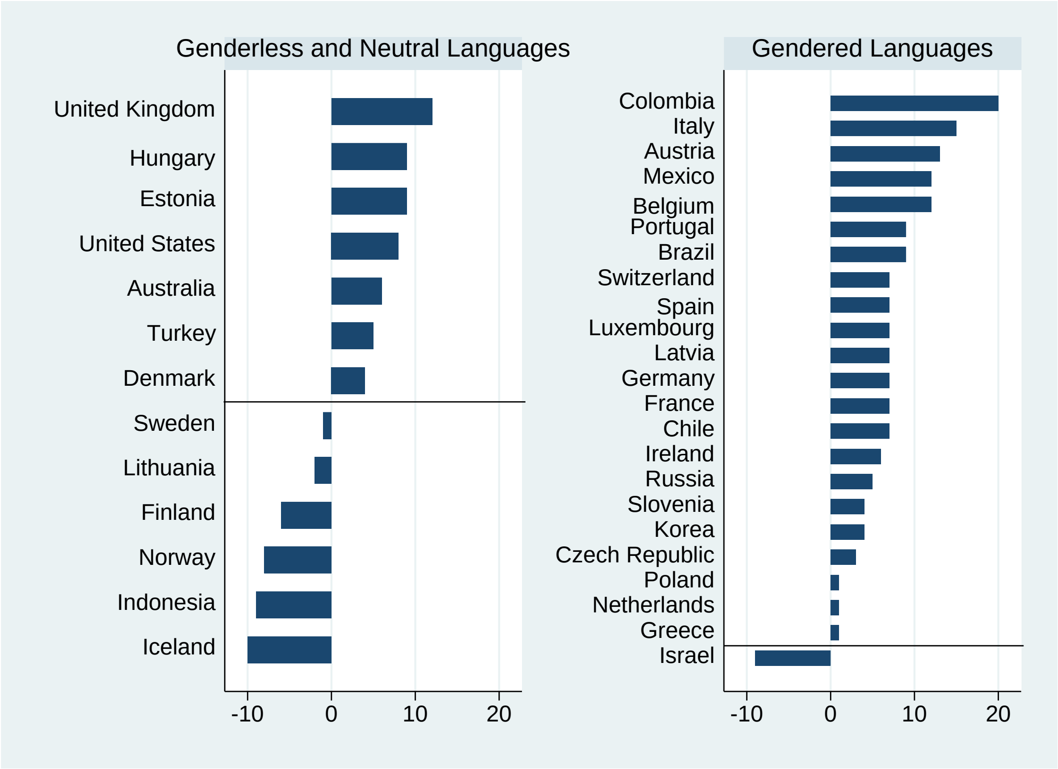

Supplementary Figure 2: The Distribution of Math Scores

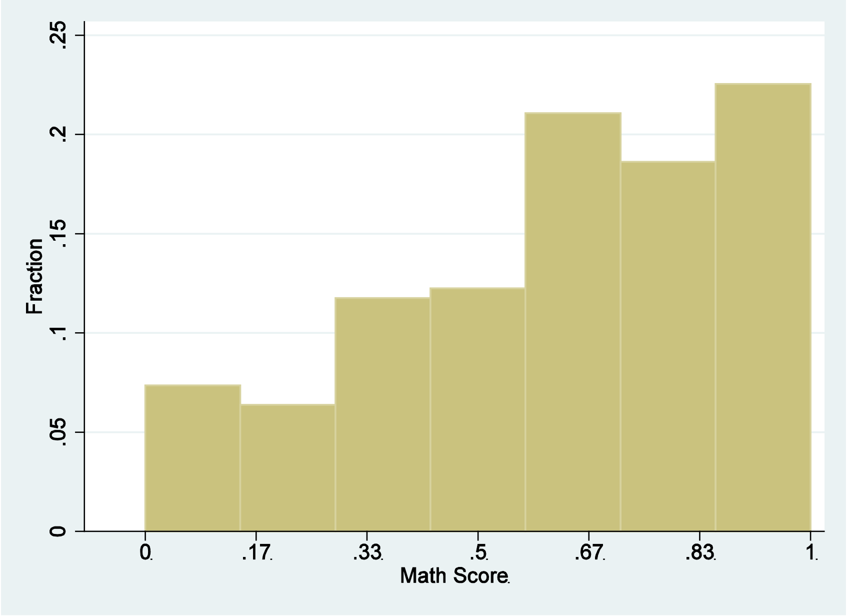

Supplementary Methods (Experimental Materials):

The math questions were taken from the website of the Israeli National Institute for Testing and Evaluation:

https://nite.org.il/files/psych/new_psych/test-hebrew.pdf.

The playwrights and philosophy reading comprehension test was taken from the Verbal Reasoning section of the July, 2016 psychometric exam:

https://www.nite.org.il/files/psych/psychometric_july_2016.pdf

The IAT and questionnaire was adopted from:

https://implicit.harvard.edu/implicit/israel/selectatest.jsp

(A) Math Test:

In the first task, you will be asked to answer several math questions. Try to answer them to the best of your ability.

Please answer (feminine/masculine form) the following question:

(1) A camel traveled for 5 days. On the first day, the camel traveled for 1 hour at a speed of 1 kilometer g per hour, and so on. How many kilometers did the camel travel during those 5 days?

1. 15
2. 20
3. 55
4. 44

Please answer (feminine/masculine form) the following question:

(2) Aladdin wishes to divide up a treasure of 19 gold coins into as many sacks as possible so that there will be a different number of coins in each sack (with each sack holding at least one coin).
How many sacks with Aladdin use?

1. 5
2. 7
3. 3
4. 8

Please answer (feminine/masculine form) the following question:

(3) Given $4 = a - 12$

$a = ?$

1. ½
2. 2
3. 1/16
4. 16

Please answer (feminine/masculine form) the following question:

(4) A jug holds 11 balls of different colors. The chance of taking a white ball out of the jug equals the chance of taking a black ball out of the jug.
Which of the following numbers could be the number of black balls in the jug?
1. 5
2. 6
3. 7
4. 8

Please answer (feminine/masculine form) the following question:

(5) Tzippi cuts 3 cucumbers in 5 minutes.
Shlomo cuts 4 tomatoes in 7 minutes.
Tzippi cut cucumbers and Shlomo cut tomatoes for 35 minutes.
How many cucumbers and tomatoes (in total) did Tzippi and Shlomo cut?
1. 60
2. 52
3. 45
4. 41

Please answer (feminine/masculine form) the following question:

(6) x, y, and z are the three interior angles of a triangle.
Given: $x = y + 10°$
$x = z + 20°$
$x = ?$

1. 55°
2. 65°
3. 70°
4. 80°

<u>(B) Empathy Reading Comprehension Test:</u>

The capacity of mankind for empathy is tremendous. However, as history shows, it is not without limits, and certainly isn't guaranteed. Researchers studying the development of empathy in children have discovered which conditions are likely to produce a compassionate and merciful person, and which lead to a person who is self-pitying, apathetic, or hostile.

Dr. Martin Hoffman from New York University suggests that empathy develops in roughly four stages, and its development ceases shortly before adolescence. In the first stage, babies have no sense of differentiation between themselves and others, and their ability to express empathy is limited to a general expression of distress when they are witnesses to distress. For example, when a nine-month-old girl sees another child fall and get hurt, she will react by burying her face in her mother's bosom.

Approximately at the age of 14 months, children begin to manifest their individuality, says Dr. Hoffman. Therefore, when that toddler sees another child who was hurt, she begins her first confused efforts to comfort him; but the boundaries between the self and others are still somewhat blurred. For example, she is likely to take the injured boy specifically to her own mother.

At the age of two, children arrive at the third stage: they distinguish the difference between the self and others. But only at the end of childhood do they express a form of empathy which is cognitive and advanced: they feel not what other people seem to feel, but rather what other people are expected to feel when given everything that is known about their condition. So, for example, if an adolescent girl sees a boy playing happily, but she also knows that this boy has cancer and is going to die soon, she, as an empathetic observer, does not feel joy.

Dr. Mark Barnett, a professor of psychology at the Kansas State University in Manhattan, suggests that the ability to care for others is dependent on one's own sense of distress of the self. Just as biologists suspect that only very intelligent animals, who are able to recognize themselves in the mirror, are able to put themselves in someone else's furry shoes, so too, children must feel secure in their own identity before they are able to relate to the needs of their surroundings.

"Children whose emotional needs have been nurtured, are better able to respond to the emotions and needs of others", says Dr. Barnett. "Children who lack self-confidence have difficulty in feeling the emotions of other people."

Beyond the question of whether they are loved, children also learn empathy by example. "Empathetic parents usually raise empathetic children" claims Dr. Barnett, "especially when the compassionate actions of the parents go beyond the immediate family. Children understand very quickly that parents who care only about their own children, really worry only for themselves.

Questions:

Please answer (feminine/masculine form) the following question:

1) In lines 21-23, the discussion of the girl who watches a boy who has cancer but is playing happily, was brought to show cases where:
    1. Children whose self-identity is not sufficiently developed, confuse their own feelings (especially when they do not wish to deal with difficult emotions such as cancer).
    2. Children find it difficult to distinguish between the feelings of joy and the feelings of sadness of others (empathetic failure).
    3. Children are able to distinguish between what people are apparently feeling as opposed to what they are expected to feel under the circumstances.
    4. Children who are able to feel the emotions of others in such an exact way, to the extent that even if the circumstances are difficult, they are able to connect to the authentic emotions of the those they see.

Please answer (feminine/masculine form) the following question:

2) The phrase "someone else" in the expression "in someone else's shoes" in line 28 relates to-
    1. Very intelligent animals
    2. Children who are in touch with their own identity
    3. Children who are not in touch with their own identity
    4. Other animals

Please answer (feminine/masculine form) the following question:

3) When a one and one-half year-old girl sees another child fall and get hurt, and in response, she begins to cry, she is expressing empathy which is appropriate to
    1. The third stage of the development of empathy (appropriate for her age) where we learn to totally distinguish between ourselves and others
    2. The fourth stage (advanced for her age) where we are able to feel complex empathy – not just what it appears that people are feeling but also what they are expected to feel
    3. The second stage (appropriate for her age) where we begin to have a sense of our own individuality
    4. The first stage (late for her age) where we feel empathy based on the expressions of general distress

Please answer (feminine/masculine form) the following question:

4) We know that the little girl that takes the wounded boy specifically to her own mother to get help, is only beginning to have a sense of her own individuality because
    1. She succeeded only partially in comforting the boy

2. She is confusing her own needs when she is hurt with the needs of the boy who was hurt
3. She is focusing on what the boy seems to be feeling but not what he is expected to feel under the circumstances
4. She is taking an example from her own mother, and not the mother of the boy who was hurt

Please answer (feminine/masculine form) the following question:

5) According to the statement: Children of parents who worry about the needs of their children and the needs of their immediate family, but not the needs of others in their surroundings
   1. Will grow up to be empathetic adults because their needs were well met when they were children
   2. Will learn from their parents and will turn out to be empathetic adults
   3. Will likely find difficulty in becoming empathetic adults, because their parents worried only for the needs of those who were close to them and not for the needs of those who were not directly close to them
   4. Will likely find difficulty in becoming empathetic adults, because their parents‘ worrying about their needs, hampers the development of their individualistic selves

(C) Playwrights and Philosophy Reading Comprehension Test:

In the first assignment, you will be asked to read a passage of text and then answer several questions related to the passage that you read. This is not meant to test your memory so the passage will be repeatedly displayed before each question.

Pierre Corneille (1606 – 1684) is considered to be one of the greatest French playwrights of all time and one of the greatest French tragedians. A tragedy is a form of theater developed in ancient Greece consisting of a rigid plot structure. The principles for writing a tragedy were outlined by the Greek philosopher Aristotle as early as the fourth century B.C.E. Debate over these guidelines was central to the discourse over literature during Corneille’s time, with two approaches being most prominent. One approach, which was conservative and purist, favored a strict adherence to the rules exactly as they were written. The other, which characterized Corneille’s approach, was more permissive and flexible. Although Corneille used Aristotle’s writings as guidelines, Corneille challenged their simple and accepted interpretation and gave them his own personal interpretation, which placed him in direct conflict with the conservative cultural establishment ruling France in his day.

In 1660, Corneille articulated his theatrical doctrine in three discourses, which could be considered a response to his critics. In one of the discourses, he responded to the claim that his plays, particularly Le Cid (1637), violated Aristotle’s principles in that they

presented an implausible plot. Although the play's plot was based on events that actually took place (or at least so they believed at the time), the critics claimed that the play was implausible—it was implausible that a decent young woman would knowingly marry her father's murderer. They believed that not every event in history could be accepted as a matter suitable for creative work and that history that was implausible was worse than subject-matter that was fictional yet plausible. However, Corneille believed that the classical demand for plausibility could actually be interpreted as a demand for integrity—the tragedy's plot did not have to be plausible, but implausible subject-matter did have to be based in history so that it would be believable to viewers. While integrity could be achieved through plausibility, it could be also be achieved through the truth.

In one article, Corneille discussed the apparent contradiction between the enjoyable and the useful. Enjoyment is the pleasure that the viewer or the reader derives from the play. Usefulness is the lesson or the moral that he learns. Many theater critics believed that these two concepts stood in conflict with one another. They thought that in order for something to be enjoyable, it had to be in some way immoral and that in order for a plot to be useful, it had to have some sort of admonishment or moral associated with it and that these qualities inherently interfered with any associated enjoyment. They claimed that anyone trying to follow Aristotle had to choose between that which was enjoyable and that which was useful, while Corneille believed that the two could be integrated. He believed that the enjoyment received from viewing a tragic play was not merely sensory, rather that it was a unique type of enjoyment stemming from the viewers' profound familiarity with the structure and principles of a tragic play. The issue at hand could be compared to a soccer game in which familiarity with the rules magnifies the enjoyment derived from watching it because the sights and sounds coming off the field thus gain significance. Such enjoyment does not involve any immorality and it is the viewer's attention that makes him open to the moral and instructive lessons interwoven into the text. If the viewers would not enjoy the play, Corneille reasoned, they would also not learn any lessons from it.

The modern reader may legitimately ask why the polemic debate over classical principles was so central to the members of that generation. The debate between Corneille and his critics must therefore be examined in light of the atmosphere prevailing in France during the seventeenth century. France in those days elevated classical Greek and Roman culture and viewed those cultures as models in all spheres of life, particularly in the arts. Against that backdrop, what level of freedom could artists living at the time adopt? That is the question that Corneille attempted to address. In large part, his refreshing interpretation and his breaching of the wall of classical principles were the first harbingers of modern theater, which completely renounced all rules and all limitations.

Please answer (feminine/masculine form) the following question:

(1) According to the passage, Corneille believed that he ______ Aristotle's principles in his plays
1. Was loyal to
2. violated only a few of
3. renounced
4. conveyed the accepted interpretation of

Please answer (feminine/masculine form) the following question:

(2) According to the second paragraph, which of the following represents the opinion of Corneille's critics regarding the plot of Le Cid?
1. It may have been implausible, but nothing prevented it from serving as the plot of a tragedy
2. It may have been fictional, but it was plausible and thus preferable to a plot that was historically true
3. While it may have been based on an event in history, it was implausible and therefore unsuitable to serve as the plot of a tragedy
4. It may have been presented as an event in history, but since it did not actually take place, it was not suitable to serve as the plot of a tragedy

Please answer (feminine/masculine form) the following question:

(3) He (highlighted in red) refers to
1. Corneille
2. The viewer or the reader
3. The theater critic
4. A follower of Aristotle

Please answer (feminine/masculine form) the following question:

(4) What was the purpose of the example of the soccer game (highlighted in red)?
1. To demonstrate the difference between enjoyment that is inherently immoral, such as the enjoyment derived from a soccer game, and enjoyment that is not inherently immoral, such as the enjoyment derived from theater
2. To clarify the claim that the understanding of the structure and principles of a tragedy could act as a source of enjoyment
3. To explain why enjoyment that requires an understanding of the rules of tragedy is also inherently useful
4. To reinforce the claim that enjoyment is actually a necessary condition for deriving usefulness

Please answer (feminine/masculine form) the following question:

(5) What is “that backdrop” (highlighted in red)?

1. The elevated status of classical culture in France during the seventeenth century
2. The debate between Corneille and his critics
3. Modern theater
4. The refreshing interpretation that Corneille brought to classical principles

Please answer (feminine/masculine form) the following question:

(6) What is a correct statement about modern theater (highlighted in red)?

1. Corneille had a role in its development
2. It was already anticipated in Corneille’s time
3. It held the writing of tragedies in high esteem
4. The principles outlined by Aristotle still served as its base

(D) Word task:

In this task you will be given a minute and asked to write (feminine/masculine form) as many words that begin with different letters. You will receive (feminine/masculine form) points for every letter of every relevant word you write (feminine/masculine form). Thus, for example, if you write (feminine/masculine form) the word 'apple' for the letter 'a' you will receive 5 points and if you write (feminine/masculine form) the word 'an' you will receive only 2.

Please follow (feminine/masculine form) the order below (do not skip (feminine/masculine form) any letter).

Write (feminine/masculine form) a word that begins with the letter A _________

Write (feminine/masculine form) a word that begins with the letter B _________

(etc.)

(E) The math and the playwright and philosophy reading comprehension tests were followed by a gender and science IAT and the following questionnaire:

Rank the level at which you link the following spheres to men and women:

Science is Choose an item.

Liberal arts and sciences are Choose an item.

What is your opinion of exact sciences Choose an item.

What is your opinion of the humanities (liberal arts and sciences) Choose an item.

How much did you enjoy studying exact sciences in school? Choose an item.

How much did you enjoy studying the humanities in school? Choose an item.

There are fewer women engineering faculty members than men. This phenomenon may sometimes be explained by the factors listed below. Please rank to what degree you believe each factor is the main reason for the phenomenon.

The average woman and the average man are different from one another in the way in which they are willing to invest time in achieving high-ranking and important positions. Choose an item.

The average woman and the average man are different in the way in which they are willing to spend time away from their families. Choose an item.

The ratio of those with a high mathematical capability is not equal among men and women (i.e. the ratio of women who are good at math is lower than the ratio of men who are good at math). Choose an item.

The average woman and the average man are different from one another in their level of interest in science. Choose an item.

The direct or indirect messages that male and female children receive do not encourage them to cultivate an interest in science in an equal manner. Choose an item.

Consciously or unconsciously, there is a tendency to favor men when recruiting and promoting employees. Choose an item.

Rank the degree to which each of the following personal goals are important to you:

To gain knowledge in science Choose an item.

To gain knowledge in math Choose an item.

To gain knowledge in liberal arts and sciences Choose an item.

Which of the following statements is most suitable to the way that you characterize yourself? Choose an item.

Original Materials (in Hebrew):

<u>(א) מבחן במתמטיקה:</u>

ענה (עני) על השאלה הבאה:

1. גמל צעד 5 ימים: ביום הראשון צעד במשך שעה במהירות 1 קמ"ש, ביום השני צעד במשך שעתיים במהירות 2 קמ"ש, ביום השלישי צעד במשך 3 שעות במהירות 3 קמ"ש, וכן הלאה. כמה ק"מ בסך הכול צעד הגמל ב-5 הימים?
   1) 15
   2) 20
   3) 55
   4) 44

ענה (עני) על השאלה הבאה:

2. אלאדין רוצה לחלק אוצר של 19 מטבעות זהב למספר רב ככל האפשר של שקים, כך שבכל שק מספר שונה של מטבעות (אך בכל שק יש לפחות מטבע אחד).
   בכמה שקים ישתמש אלאדין?
   1) 5
   2) 7
   3) 3
   4) 8

ענה (עני) על השאלה הבאה:

3. נתון: $a-12=4$

   $a = ?$

   1) 1/2
   2) 2
   3) 1/16
   4) 16

ענה (עני) על השאלה הבאה:

4. בכד 11 כדורים בצבעים שונים. הסיכוי להוציא מהכד כדור לבן שווה לסיכוי להוציא מהכד כדור שחור.
   איזה מן המספרים הבאים יכול להיות מספר הכדורים השחורים בכד?
   1) 5
   2) 6
   3) 7
   4) 8

ענה (עני) על השאלה הבאה:

5. ציפי חותכת 3 מלפפונים ב-5 דקות.

שלמה חותך 4 עגבניות ב-7 דקות.

במשך 35 דקות, ציפי חתכה מלפפונים ושלמה חתך עגבניות.

כמה מלפפונים ועגבניות (בסך הכול) חתכו ציפי ושלמה?

1) 60
2) 52
3) 45
4) 41

ענה (עני) על השאלה הבאה:

6. x, y ו-z הן שלוש הזוויות הפנימיות במשולש.

נתון: $x = y + 10°$

$x = z + 20°$

$x = ?$

1) 55°
2) 65°
3) 70°
4) 80°

<u>(ב) קטע קריאה בנושא אמפתיה</u>

קרא (קראי) בבקשה את קטע הקריאה וענה (עני) על השאלות שבסופו:

היכולת האנושית לאמפתיה אדירה, אך, כפי שמבהירה ההיסטוריה, היא אינה חסרת תחתית ואף לא מובטחת. חוקרים המתבוננים בהתפתחות אמפתיה בילדים מצאו אילו תנאים עשויים להניב אדם חנון ורחום, ואילו אדם מרחם על עצמו או אדיש או עוין.

ד"ר מרטין הופמן מאוניברסיטת ניו יורק מציעה כי אמפתיה מתפתחת בערך בארבעה שלבים, והתפתחותה מסתיימת רק מעט לפני גיל ההתבגרות. בשלב הראשון אין לתינוקות תחושה של הפרדה בין העצמי לאחרים, ויכולתם להבעת אמפתיה מוגבלת להבעה כללית של מצוקה כאשר הם עדים למצוקה. כשילדה בת תשעה חודשים, למשל, רואה ילד נוסף נופל ונפצע, היא תגיב על ידי כך שתקבור את פניה בחיקה של אמה.

בגיל 14 חודשים בערך, ילדים מתחילים לשלוט באינדיווידואליות שלהם אומרת ד"ר הופמן. לכן, כשפעוטה רואה ילד אחר שנפגע, היא מתחילה בניסיונות המביכים הראשונים שלה לנחם אותו; אבל הגבולות בין העצמי ואחרים עדיין מטושטשים מעט, והפעוטה עשויה, למשל, להוליך את הילד שנפגע דווקא לאמה לעזרה.

בגיל שנתיים מגיעים הילדים לשלב השלישי: הם מזהים את ההבדל בין העצמי לבין אחרים. אבל רק בסוף הילדות, הם מביעים צורה אמפטית קוגניטיבית מתוחכמת: הם מרגישים לא מה שנראה שאנשים אחרים מרגישים, אלא מה שאנשים אחרים אמורים להרגיש בהנתן כל מה שידוע על מצבם של אלו. כך, למשל,

אם נערה רואה ילד משחק בשמחה, אבל היא גם יודעת שלילד יש סרטן ושהוא עומד למות בקרוב, היא כצופה אמפתית לא מרגישה שמחה.

ד"ר מארק ברנט, פרופסור לפסיכולוגיה באוניברסיטת קנזס סטייט במנהטן, מציע כי האפשרות לטפל באחרים תלויה בתחושה מוצקה של העצמי. בדיוק כפי שביולוגים חושדים כי רק בעלי חיים אינטליגנטיים מאוד אשר מסוגלים להכיר את עצמם במראה יכולים לשים את עצמם בנעליים הפרוותיות של האחר, כך ילדים חייבים להרגיש בשליטה על הזהות שלהם לפני שהם יכולים להתייחס לצרכים של הסביבה. "ילדים אשר הצרכים הרגשיים שלהם מטופלים, יכולים להענות יותר לרגשות ולצרכים של אחרים", אומר ד"ר ברנט. "לילדים חסרי ביטחון יש קושי לחוות רגשות של אנשים אחרים".

מעבר לשאלה האם הם אהובים, ילדים לומדים אמפתיה גם בדרך של דוגמה. הורים אמפתים בדרך כלל מגדלים ילדים אמפתים, אומר ד"ר ברנט, בייחוד כשהמחוות החומלות של ההורים מתרחבות מעבר לבני המשפחה הקרובה. ילדים מבינים די מהר כי הורים שלהם אכפת רק מצאצאיהם, באמת דואגים רק לעצמם.

<u>שאלות</u>

1. ענה (עני) על השאלה הבאה:

בשורות 15-17, הדיון בילדה הצופה בילד החולה בסרטן ומשחק בשימחה נערך על מנת להדגים מצבים שבהם:

1) ילדים שהעצמי שלהם אינו מפותח דיו ולכן הם מבלבלים בין רגשותיהם (במיוחד כשאינם רוצים להתמודד עם רגשות קשים כמו מחלת הסרטן).
2) ילדים המתקשים להבחין בין רגשות השימחה לרגשות העצב של אחרים (כשל אמפתי).
3) ילדים המצליחים להבחין בין מה שנראה שאנשים מרגישים לבין מה שהם אמורים להרגיש בנסיבות.
4) ילדים שמצליחים להרגיש את הרגשות של אחרים בצורה כל כך מדוייקת עד שגם אם הנסיבות קשות, הם מצליחים להתחבר לרגש האותנטי של מי שהם רואים אותו.

2. ענה (עני) על השאלה הבאה:

"האחר" בביטוי "בנעליים הפרוותיות של האחר" בשורה 20 מתייחס ל –

1) בעלי חיים אינטליגנטיים מאוד
2) ילדים שמרגישים בשליטה
3) ילדים שאינם מרגישים בשליטה
4) בעלי חיים אחרים

3. ענה (עני) על השאלה הבאה:

כשילדה בת שנה וחצי רואה ילד נוסף נופל ונפצע ובתגובה מתחילה לבכות היא מביעה אמפתיה שמתאימה:

1) לשלב השלישי בהתפתחות האמפתיה (המתאים לגילה) שבו אנחנו לומדים להבחין בינינו לבין אחרים באופן מלא
2) לשלב הרביעי (המתקדם לגילה) שבו אנחנו מצליחים להרגיש אמפתיה מורכבת – לא רק מה שנראה שאנשים מרגישים אלא גם למה שהם אמורים להרגיש
3) לשלב השני (המתאים לגילה) שבו אנחנו מתחילים לשלוט באינדיבידואליות שלנו
4) לשלב הראשון (המאוחר לגילה) שבו אנחנו מרגישים אמפתיה שמתבטאת בהבעת מצוקה כללית

4. ענה (עני) על השאלה הבאה:

הפעוטה שמוליכה את הילד שנפגע דווקא לאמה שלה לעזרה מבטאת רק את תחילתה של שליטה באינדיבידואלייות שלה משום ש .

1) היא מצליחה לנחם את הילד רק באופן חלקי
2) היא מבלבלת בין הצורך שלה כשהיא נפגעת לצורך של הילד שנפגע
3) היא מתמקדת במה שנראה שהילד מרגיש אבל לא במה שהוא אמור להרגיש בנסיבות האלה
4) היא מקבלת דוגמא מאמה שלה ולא מהאמא של הילד שנפגע

5. ענה (עני) על השאלה הבאה:

לפי הקטע, ילדים להורים שדואגים לצריכהם של ילדיהם ושל כל צרכי המשפחה הקרובה שלהם, אך לא לצרכיהם של אחרים בסביבתם

1) יגדלו להיות מבוגרים אמפתיים משום הצרכים שלהם נענו בצורה טובה כשהיו ילדים
2) ילמדו מהוריהם ויהפכו להיות מבוגרים אמפתיים
3) עשויים להתקשות להפוך ולהיות מבוגרים אמפתיים, כי הוריהם דאגו רק לצרכים של מי שקרוב אליהם ולא לצרכיהם של מי שאינו קשור אליהם באופן מיידי
4) עשויים להתקשות ולהפוך להיות מבוגרים אמפתיים כי הדאגה של ההורים שלהם לצרכיהם מקשה על התפתחות העצמי האינדיווידואלי שלהם

<u>(ג) קטע קריאה בנושא מחזאים ופילוסופיה</u>

במשימה הראשונה אתה (את) מתבקש (מתבקשת) לקרוא קטע קריאה ולאחר מכן לענות על מספר שאלות המתייחסות לקטע שקראת. המשימה היא לא משימת זיכרון ולפיכך אותו קטע קריאה יוצג שוב ושוב לפני כל שאלה.

פייר קורניי (1606-1684) נחשב אחד המחזאים הצרפתים הדגולים בכל הזמנים וגדול כותבי הטרגדיה הצרפתים. הטרגדיה היא סוגת תאטרון בעלת מבנה עלילתי נוקשה שהתפתחה ביוון הקדומה, ואת העקרונות לכתיבתה התווה הפילוסוף היווני אריסטו כבר במאה הרביעית לפני הספירה. בימיו של קורניי תפס הדיון בעקרונות אלו מקום מרכזי בשיח הספרותי, ובלטו בו שתי גישות. האחת, שמרנית וטהרנית, דגלה בהקפדה יתרה על העקרונות ככתבם וכלשונם. האחרת, גישתו של קורניי, הייתה מתירנית וגמישה יותר. אף שכתביו של אריסטו היו נר לרגליו של קורניי, הוא קרא תיגר על הפרשנות הפשוטה והמקובלת להם ופירשם פירוש אישי, והדבר העמידו בעימות ישיר מול הממסד התרבותי השמרני ששלט בצרפת בימיו.

את עיקר משנתו בתחום התאטרון כינס קורניי בשנת 1660 בשלושה מאמרים, ואפשר לראות בהם תגובה למבקריו. באחד המאמרים הוא השיב לטענה כי במחזותיו, ובמיוחד ב"לה סיד" (1637), חטא לעקרונות של אריסטו בהציגו עלילה בלתי-סבירה. אף על פי שעלילת המחזה מתבססת על אירועים שהתרחשו באמת (או כך לפחות האמינו בזמנו), טענו המבקרים שהיא אינה מתקבלת על הדעת: אין זה סביר שנערה מכובדת תינשא ביודעין לרוצח אביה. לדידם, לא כל אירוע היסטורי יכול להתקבל על הדעת בתור נושא ליצירה, והיסטוריה בלתי-סבירה גרועה מנושא מומצא אך סביר. ברם, קורניי גרס שאפשר לפרש את הדרישה הקלאסית לסבירות כדרישה לאמינות דווקא: אין הכרח שעלילת הטרגדיה תהיה סבירה, אך מה שאינו מתקבל על הדעת חייב להיות מעוגן בהיסטוריה כדי שהצופים יאמינו בו. אמנם אפשר להשיג אמינות בעזרת סבירות, אך אפשר להשיגה גם בעזרת האמת.

במאמר אחר דן קורניי בניגוד שיש לכאורה בין המהנה ובין המועיל. ההנאה היא העונג שהצופה או הקורא מפיק מן המחזה. התועלת היא הלקח או המוסר שהוא לומד. מבקרי תאטרון רבים סברו ששני המושגים האלה עומדים בסתירה זה לזה: כדי להסב הנאה יש לגרום בהכרח להשחתה כלשהי של המידות, וכדי להפיק תועלת יש להשמיע דברי תוכחה ומוסר, ואלה פוגמים בהנאה הצרופה. הם טענו שעל ההולך בדרכו של אריסטו לבחור בין המהנה למועיל, ואילו קורניי חשב שאפשר לשלב בין השניים. לדעתו, ההנאה מהמחזה הטרגי אינה הנאה חושית גרידא, אלא הנאה ייחודית הנובעת מהיכרותם העמוקה של הצופים עם המבנה והעקרונות שהטרגדיה כפופה אליהם. למה הדבר דומה? למשחק הכדורגל, שהיכרות עמוקה עם כלליו מעצימה את ההנאה ממנו, משום שהיא המעניקה משמעות למראות ולקולות על המגרש. בהנאה כזאת אין כל השחתה של המידות, ובמושכה את לב הצופה היא עושה אותו קשוב לדברי המוסר והלקח השזורים בטקסט. אם הצופים לא ייהנו מן המחזה, פסק קורניי, הם גם לא יפיקו ממנו תועלת.

בצדק ישאל את עצמו הקורא המודרני מדוע היה הפולמוס על העקרונות הקלאסיים מרכזי כל כך בעיני בני התקופה. ובכן, את הדיון בין קורניי למבקריו יש לבחון לנוכח הלך הרוח ששלט בצרפת במאה השבע עשרה. צרפת של הימים ההם רוממה את התרבויות הקלאסיות, היוונית והרומית, וראתה בהן דוגמה ומופת בכל תחומי החיים,ובמיוחד באמנויות. מה מידת החירות שיוצר בן אותו הזמן היה יכול ליטול לעצמו על הרקע הזה? זו השאלה שקורניי עסק בה. במידה רבה, הפרשנות המרעננת שהביא והסדקים שהבקיע בחומת העקרונות הקלאסיים היו הסנונית הראשונה למגמות המודרניות בתאטרון, המתנערות לחלוטין מכל כלל ומכל מגבלה.

ענה (עני) בבקשה על השאלה הבאה:

(1) על פי הקטע, קורניי חשב שבמחזותיו הוא ______ עקרונותיו של אריסטו

1. היה נאמן ל-
2. הפר רק כמה מ-
3. התנער מ-
4. הביא לידי ביטוי את הפרשנות המקובלת ל-

ענה (עני) בבקשה על השאלה הבאה:

(2) על פי הפסקה השנייה, איזו מהקביעות הבאות מייצגת את דעת מבקריו של קורניי בנוגע לעלילת המחזה לה סיד?

1. אמנם אין היא סבירה, אך אין מניעה שהיא תהיה עלילת טרגדיה
2. אמנם היא בדויה, אך היא סבירה ולכן עדיפה מעלילה היסטורית
3. אמנם היא מבוססת על אירוע היסטורי, אך מאחר שאינה סבירה, היא אינה ראויה להיות עלילת טרגדיה
4. אמנם היא מוצגת כאירוע היסטורי, אך מאחר שלא התרחשה באמת, היא אינה ראויה להיות עלילת טרגדיה

ענה (עני) בבקשה על השאלה הבאה:

(3) הוא (מודגש באדום), כלומר –
1. קורניי
2. הצופה או הקורא
3. מבקר התאטרון
4. ההולך בדרכו של אריסטו

ענה (עני) בבקשה על השאלה הבאה:

(4) מה תפקידה של דוגמת משחק הכדורגל (מודגש באדום)?
1. להראות את ההבדל בין הנאה הכרוכה בהשחתת המידות, כמו ההנאה ממשחק כדורגל, לבין הנאה שאינה כרוכה בהשחתת המידות, כמו ההנאה מתאטרון
2. להבהיר את הטענה שהבנת מבנה הטרגדיה ועקרונותיה יכולה להיות מקור להנאה
3. להסביר מדוע הנאה המחייבת הבנה של כללי הטרגדיה טומנת בחובה גם תועלת
4. לחזק את הטענה שלמעשה ההנאה היא תנאי הכרחי להפקת תועלת

ענה (עני) בבקשה על השאלה הבאה:

(5) מהו "הרקע הזה" (מודגש באדום)?
1. המעמד הרם של התרבות הקלאסית בצרפת של המאה השבע עשרה
2. הפולמוס שהתנהל בין קורניי למבקריו
3. המגמות המודרניות בתאטרון
4. הפרשנות המרעננת שהביא קורניי לעקרונות הקלאסיים

ענה (עני) בבקשה על השאלה הבאה:

(6) מה נכון לומר על המגמות המודרניות בתאטרון (מודגש באדום)?
1. לקורניי היה תפקיד בהתפתחותן
2. הן היו צפויות כבר בימיו של קורניי
3. הן מעלות על נס כתיבת טרגדיות
4. בבסיסן עדיין עומדים העקרונות שהתווה אריסטו

<u>(ד) משימת מילים:</u>

במשימה זו תתבקש (תתבקשי) לכתוב במשך דקה כמה שיותר מילים שמתחילות באותיות מסוימות. תקבל (תקבלי) ניקוד על כל מילה נכונה ולפי מספר האותיות שלה (נקודה לכל אות). כך למשל, אם תכתוב (תכתבי) את המילה תפוח באות ת תקבל (תקבלי) 4 נקודות ואם תכתוב (תכתבי) את המלה תם תקבל (תקבלי) רק 2 נקודות על מילה זו.

אנא כתוב (כתבי) את המילים לפי הסדר (אל תדלג (תדלגי) על אותיות).

בהצלחה!

כתוב (כתבי) מילה שמתחילה באות א: ________________

כתוב (כתבי) מילה שמתחילה באות ב: ________________

כתוב (כתבי) מילה שמתחילה באות ג: _______________

(וכו'...)

(ה) לאחר המבחן במתמטיקה וקטע הקריאה בנושא מחזאים ופילוסופיה ניתן מבחן IAT בנושא מגדר ומדעים ולאחריו השאלון הבא:

דרגו עד כמה אתם מקשרים בין התחומים הבאים לגברים ולנשים

מדע

מדעי הרוח ואומנות

מה העמדה שלך לגבי מדע מדויק

מה העמדה שלך לגבי מקצועות הומניים (מדעי הרוח ואומנות)?

מנסיונך בלימודים, עד כמה אהבת ללמוד מדע מדויק?

מנסיונך בלימודים, עד כמה אהבת מקצועות הומניים?

יש פחות נשים מגברים במשרות אקדמאיות בפקולטות להנדסה. התופעה מוסברת לפעמים בעזרת הגורמים שיפורטו להלן. דרג\י בבקשה עד כמה כל גורם נראה לך כסיבה מרכזית לתופעה.

האישה הממוצעת והגבר הממוצע שונים זה מזה במידה שבה הם מוכנים להשקיע מזמנם כדי לזכות במשרות גבוהות וחשובות.

האישה הממוצעת והגבר הממוצע שונים במידה שבה הם מוכנים לבלות זמן מחוץ לחיק המשפחה.

שיעור בעלי יכולת גבוהה במתמטיקה אינו שווה בקרב נשים וגברים (כלומר, שיעור הנשים שטובות מאוד במתמטיקה נמוך משיעור הגברים שטובים במתמטיקה).

האישה הממוצעת והגבר הממוצעים שונים זה מזה במידת העניין שלהם במדע.

המסרים הישירים או העקיפים שילדים וילדות מקבלים אינם מעודדים אותם במידה שווה לטפח עניין במדע.

במודע או שלא במודע, ישנה נטייה להעדיף גברים בגיוס ובקידום עובדים.

דרג\י עד כמה כל אחת מהמטרות האישיות הבאות חשובה לך:

לצבור ידע במדע

לצבור ידע במתמטיקה

לצבור ידע במדעי הרוח ואומנות

איזו מהאמירות הבאות הכי מתאימה להגדרה העצמית שלך?
